# Role of non-governmental organizations in mental health in India

**DOI:** 10.4103/0019-5545.69276

**Published:** 2010-01

**Authors:** R. Thara, Vikram Patel

**Affiliations:** Schizophrenia Research Foundation, Chennai, India; 1 London School of Hygiene and Tropical Medicine and Sangath, Goa, India

**Keywords:** India, mental health, role of NGOs

## Abstract

The paucity of treatment facilities and psychiatrists in the Government sector has widened the treatment gap in mental health. Non-governmental organizations (NGOs) have played a significant role in the last few decades in not only helping bridge this gap, but also by creating low cost replicable models of care. NGOs are active in a wide array of areas such as child mental health, schizophrenia and psychotic conditions, drug and alcohol abuse, dementia etc. Their activities have included treatment, rehabilitation, community care, research, training and capacity building, awareness and lobbying. This chapter outlines the activities of NGOs in India. This is a revised version of the chapter in the book on mental health to be brought out by Government of India.

## INTRODUCTION

Mental health has for decades been low in the priority of health planners at state and central levels and this is well reflected in the quantity and quality of mental health services in India. The needs of patients and families far outstrip the availability and accessibility of services for those with mental disorders. India’s scarce mental health resources, such as mental health specialists, are largely concentrated in some states (mainly in the south) and in urban areas and a large proportion are solely in the private sector. Over half of all inpatient beds are located in 40 odd mental hospitals, most of which were built during the colonial years. It is not surprising, then, that the ‘treatment gap’ for mental disorders is large all over the country, but especially so in rural areas, northern states and amongst the socially disadvantaged.

While the government or public services are the key providers of care for these populations, and therefore need strengthening, the NGO movement in the country has seen a steady upswing in the last two decades to fill the large gaps. NGOs are driven by a passion towards a certain cause and back it up with commitment and drive. While the reach of their work cannot parallel that of government agencies, the quality of care and their efforts in reaching out to the various stakeholders, particularly those who are discriminated against such as persons with mental disorders, gives them a distinct advantage.

This chapter seeks to provide an overview of the contributions of Mental Health NGOs (MHNGOs) in India. A brief profile of some NGOs working in key and distinct areas should enable the readers to understand better the ways in which NGOs can innovate, replicate and complement state run services.

## HISTORICAL ASPECTS

Non-Governmental Organizations (NGOs) are institutions, recognized by governments as non-profit or welfare oriented, which play a key role as advocates, service providers, activists and researchers on a range of issues pertaining to human and social development. Historically, NGOs have played a critical role in promoting and facilitating health and educational activities in India. Prior to independence, religious bodies set up a number of educational institutions, health facilities and other charities. These movements were often led by charismatic individuals, driven by a sense of missionary zeal. Many NGOs were born in response to major disasters and crises with the aim of providing emergency relief and rehabilitation. Since independence, there has been a meteoric rise in the profile, breadth and range of NGOs in the country.

Three key changes have occurred in the evolution of the NGO- first, the greater degree of professionalization of NGO activities; second, the widening of sources of funds for NGOactivities to include major national and international donor agencies; and third, the secular origins of NGOs. The growing professionalization of NGOs led to the evolution, in the 1960s, of NGOs which focused on health issues. These NGOs increasingly filled gaps in healthcare provision, focusing on under-served populations. Some of these NGOs have now become large institutions in their own right, providing primary care services and strengthening community action for change. The activities of, now internationally acclaimed, NGOs such as the Self Employed Women’s Association (SEWA), the Karuna Trust and the Aravind Eye Care group, have become models for wider adoption by the government in its own program development. Much has already been written and documented on the work of NGOs in a variety of sectors of community development issues, including health (Pachauri 1994).[1] However, there had been no such initiative in the specific area of mental health until the recent documentation of a number of NGO programs in mental health in India by the authors (Patel and Thara, 2003).[2] This chapter is based on this recent report.

## DIVERSITY OF MENTAL HEALTH NON-GOVERNMENTAL ORGANIZATIONS

Despite the considerable challenges faced in developing mental health programs, it is gratifying to note the achievements made by many MHNGOs are distributed throughout the country, although there are a greater number in urban areas, and in states where there are relatively lesser pressing problems posed by poverty and communicable diseases (for example, southern states). In part, this is because these areas already have the mental health specialist resources that are often critically important in leading the development of NGO-based services. Another reason is that these are the areas of the country where the epidemiological transition is more advanced and where mental disorders account for a larger proportion of the burden of disease. Although MHNGOs are predominantly urban in location, many have begun to extend services into rural areas. Most MHNGOs serve a defined community; however, the work of some has spread to more than one center or geographical region. Examples of such NGOs are the Alzheimer and Related Disorders Society of India (ARDSI), which was started in Cochin, and has now spread to more than a dozen centers in India. Similarly, the Richmond Fellowship Society has three centers. The oldest MHNGOs in India are probably those working in the field of child mental health, and in particular, mental retardation. This may not be surprising given the close nature of the relationship between mental retardation and the concept of childhood disabilities which has been one of the bedrocks of the NGO movement for several decades. The concept of child mental health has broadened from its earlier focus on mental retardation to include the far commoner mental health problems seen in children, such as autism, hyperactivity and conduct disorders. MHNGOs such as Sangath Society (Goa) and Umeed and the Research Society (Mumbai) provide outpatient and school based services for such problems.

Other than mental retardation, the other early MHNGOs had care and treatment and rehabilitation as their priorities and developed appropriate models of rehabilitation in diverse settings and for diverse clinical populations. Their primary focus was on severe mental disorders and many of these MHNGOs (such as the Schizophrenia Research Foundation (SCARF) in Chennai, Manas in West Bengal, the Medico-Pastoral Association (MPA) in Bangalore, and Shristi in Madurai were started by psychiatrists who already held full-time faculty positions in the local medical school. These MHNGOs were started to fulfill the need for a broader, holistic approach to the management of severe mental disorders. Thus, activities ranging from family counseling to vocational rehabilitation, which were rarely provided in psychiatric out-patient clinics, were given greater attention. Another area of mental health which attracted considerable interest and attention was substance abuse. Alcohol abuse and, in particular, drug abuse captured the public imagination and received considerable media interest in the 1970s and 80s. This public attention and the obvious need for community-based rehabilitation services for persons affected by substance abuse led to the development of numerous MHNGOs working in this area. The TTK Hospital in Chennai, the TRADA in Karalla and Karnataka, Parivarthan in Maharashtra, Kripa Foundation, Alcoholics Anonymous and the Samaritans in many parts of the country and the National Addiction Research Center in Mumbai are examples of MHNGOs focusing on substance abuse problems.

National programs on alcohol and drug abuse are increasingly being implemented through grants-in-aid to such NGOs. More recently, the scope of activities of MHNGOs has broadened further, with a better understanding of the range and nature of mental health problems. Thus, stress-related disorders such as anxiety and depression are increasingly recognized as major causes of sickness and disability. MHNGOs providing community based counseling and suicide prevention activities have mushroomed. Reports highlighting the rising rates of suicide in India, in particular amongst young people, have alerted health professionals and the community about this serious mental health problem. Sneha (Chennai), MPA (Bangalore) and Saarthak (Delhi) work on suicide prevention activities; many NGOs now run help-lines for distressed persons. Some MHNGOs focus on women’s mental health; common mental disorders, which are often linked to stress and oppression, are not surprisingly, more frequent in women. The activities of the Bapu Trust (Pune) demonstrate how the feminist theory can contribute to the discourse on the linkages between women’s lives in a gender-biased society and their mental health. Banyan (Chennai) provides shelter and care for women living with mental disorders.

Two welcome developments in the NGO sector are the growth in user/family NGOs and the inclusion of mental health by NGOs whose original mandate was in other areas of health. Some MHNGOs, such as ACMI (Bangalore) and Aasha in Chennai are entirely run by, and focus on, families of those affected by severe mental disorders. ARDSI works with families who have a member affected by dementia. The growth of these, non-professional, family oriented MHNGO sector is to be welcomed for it is very likely that the needs of the mentally ill may be expressed and met in different ways by families and by mental health professionals. Basic Needs is an NGO which combines both service, delivery with an emphasis on livelihood skills development to empower people with mental disorders. Adolescent health interventions based on the life skills model, have become very popular in secondary schools around the country; many such programs are run in collaboration with local NGOs focusing on reproductive and sexual health issues. Targeted interventions for injectable drug users are also supported though national programs for HIV/AIDS (NACO) and implemented through NGOs focusing on HIV/AIDS (for e.g. Positive People in Goa). Many disability-focused NGOs now include mental health as a core element. An example of such an MHNGO is Ashagram in Madhya Pradesh whose primary focus was physical disabilities, especially persons affected by leprosy but which expanded it community based rehabilitation program to include severe mental disorders which also produce a profound disability in some persons. Other examples of broad-based NGOs which are integrating mental health in their agenda include the Voluntary Health Associations of India and the Community Health Cell (Bangalore). These are healthy trends facilitating the view of mental health as an integral component of the broader rubric of public health.

Despite considerable diversity in the range of objectives and types of MHNGOs as described above there are several common features shared by many of the MHNGOs. The perceived need of the community appears to have been a major catalyzing factor for the initiation and sustainability of all the MHNGOs. In some cases, personal tragedies and first hand experiences have been inspirational factors. Scepticizm and cynicism, especially of the medical community, and lack of cooperation and sensitivity of government officials and donor agencies have been uniform experiences, especially in the founding years. Not unexpectedly, a high premium is placed on involvement of families and other stakeholders in the activities and programs of all the MHNGOs. For many MHNGOs, government funding support is minimal; and most are dependant on general public or donor agencies for financial resources. A few have been able to mobilize research funds, by virtue of having established research credentials. Many MHNGOs charge fees for services. Let us now consider the kinds of activities which MHNGOs are engaged in working towards their objectives towards improving the health of those affected by mental disorders.

## MENTAL HEALTH NON-GOVERNMENTAL ORGANIZATIONS ACTIVITIES AND PROGRAMS

We have grouped the activities of the MHNGOs in the following broad categories for the sake of discussion; however, there are obvious overlaps between some of these activities:

1. Treatment: care and rehabilitation
2. Community-based activities and prevention
3. Research and training
4. Advocacy and empowerment

### Treatment: Care and rehabilitation

It was natural for many MHNGOs to identify treatment and rehabilitation as their priorities, based on the felt and largely unmet needs of the populations they wished to serve. Models of care and rehabilitation have been developed, many of which are replicable in diverse settings. While most state-run organizations focus on medical treatment, psycho-social rehabilitation (PSR) is sadly a neglected though major aspect of MHNGO programs. The absence of trained staff to carry out PSR activities has, however, kept it away from mainstream psychiatric services. Hence, many NGOs have taken it upon themselves to develop modules of PSR in both urban and rural areas. The programs include a spectrum of activities such as individual and group counseling, vocational rehabilitation and livelihood skills training, cognitive retraining, family support and counseling, self-help groups, recreation and leisure activities. The range of care facilities depends on the conditions which are the focus and the resources of individual NGOs. Out-patient clinics, in-patient care, day care programs and long term residential care form the spectrum of services provided by MHNGOs, especially the ones dealing with chronic psychotic conditions. Within this spectrum of services, a range of treatments including drug and psychological treatments are offered. Many persons require long-term care to minimize the disability associated with some mental disorders such as schizophrenia and dementia. Typically, about a third of patients with schizophrenia will show signs of long-term disability associated with a variety of factors such as chronic symptoms, stigma and the side effects of medication. Most MHNGOs working in this area have comprehensive services focusing both on the control of symptoms of the acute phase of the illness, as well as rehabilitation to ensure optimal functioning in the longer-term. Providing vocational training in skilled professions such as carpentry and printing, social skills training and family therapy are some examples of the kind of activities undertaken. MHNGOs provide linkages with potential employment by sensitizing employers to the needs of those suffering from chronic mental disorders.

Specific interventions targeted to groups such as children or the elderly are also being offered by some MHNGOs. In the case of child mental health, for example, interventions targeted at children, their parents and class room interventions are offered. Childhood mental disorders also require a range of rehabilitation interventions, particularly in the educational field. MHNGOs working in other areas, such as substance abuse, also provide a range of rehabilitation services.

### Community programs and prevention

Although the National Mental Health Program was initiated in 1983 to ensure minimum standards of mental health care by integration with existing primary healthcare services, this still remains a utopian dream in almost all parts of the country. A major reason for this is the almost complete biomedical emphasis of the program with an outpatient clinic where medicines are doled out in a health centre being the principal and, indeed in most places, the only form of care which is provided. On the other hand, NGOs have initiated a number of community-based mental health programs emphasizing on services in a variety of community, including home-based, settings and offer a range of PSR activities. These programs range from primary prevention activities such as suicide prevention (see below) to provision of treatment in community clinics, increasing awareness and providing community based rehabilitation (CBR). NGOs are arguably better placed to approach and win the trust of local communities, establish ties with them and locate their programs in and for the community. Examples of primary prevention programs are the telephone help lines for depressed and suicidal persons, early intervention for babies born at risk for developmental delay and education programs in schools and workplaces for prevention of substance abuse.

Secondary prevention focuses on minimizing the handicaps associated with an existing mental disorder. Examples of such programs include CBR programs for child and adult mental disabilities and school programs to help children with hyperactivity and dyslexia stay in school. CBR is an essential ingredient of community care programs. SCARF, as part of vocational support activities, has distributed livestock, cows and helped expansion of petty shops in rural areas to help persons with schizophrenia. This is not just a means of livelihood, but has also improved their functioning and involvement in many ways. Empowerment of the local community is equally important and involvement of key and influential persons in the community such as teachers, religious heads, and local administrators has yielded good results. Basic Needs is another MHNGO which emphasizes on such CBR activities as the core component of its mental health program. Community programs gained much significance when the tsunami left in its wake a number of psycho-social problems which required intensive counseling, support and sometimes medication to allay anxiety and depression. NGOs like SNEHA (Chennai) provided a range of community based counseling and mental health interventions in the aftermath of this disaster. In keeping with latest advances in technology and communications, SCARF has started using telemedicine to expand access to specialist mental health services in rural areas. Homelessness and the destitute mentally ill have also received growing attention in the last decade or so. NGOs such as Banyan and Anbagam in Chennai, Ashadeep in Guwahati and Samarpan in Indore, and a few others, have developed comprehensive services for the “wandering” mentally ill. However, to sustain these programs, a national plan is required for the provision of care to the homeless and wandering mentally ill, whose plight is borne out of a combination of health, socio-economic and human rights issues.

In the 1970s and 80s, there was a mushrooming of NGOs to deal with substance abuse, but few of these organizations have sustained themselves. The TTK hospital/TT Ranganathan Clinical Research Foundation started in 1980 in Chennai is an organization which has expanded itself in various activities and become a referral center for training and awareness building in substance abuse. This NGO has been active in the various fields of out-patient and in-patient care, extending care to those who need it through community outreach programs and CBR activities.

The Indian Alcohol Policy Alliance, a network of centers and individuals working in de-addiction have released an” “Alcohol Atlas of India” as a reference guide for policy makers and professionals The National Addiction Research Centre (NARC), established in 1985, is another example of an NGO focusing on substance abuse who have sustained their activities, in part due to the growing emphasis on drug users as a target population for HIV/AIDS control.

### Research and training

Until relatively recently, MHNGOs were primarily concerned with service provision and advocacy related activities. Research was considered as an academic exercise, best reserved for the ivory towers of universities and teaching hospitals. This has changed so much in recent years that today MHNGOs are at the forefront of ground-breaking health research in India. Major research programs in health areas as diverse as infectious diseases to nutrition are now conducted under the aegis of NGOs. MHNGOs are no exception to this trend. The SCARF studies on schizophrenia are the most widely-cited research on the subject from any developing countries (Thara and McCreadie 1998).[3] All three published studies of dementia in the community in India are from work done by MHNGOs (REF). Sangath’s studies on the treatment of depression are amongst the largest such studies from India (Patel *et al*. 2003),[4] Sangath’s Manas project is the largest trial for a mental health treatment from any developing country. Ashagram’s community program for schizophrenia has generated the first scientific evidence of the use of the CBR approach for rehabilitation of a mental disorder (Chatterjee *et al*. 2003).[5] The experience of CBR in Ashagram has led to the initiation of the first randomized controlled trial of this approach in three sites in India. These are just some examples of innovative, action-oriented research emanating from MHNGOs.

Many MHNGOs actively invest in the development of skills of their staff and of other stakeholder groups. Participation in workshops, conferences and seminars, and formal training in courses such as rehabilitation are often offered as opportunities for career development. Most of the MHNGOs provide opportunities for training other professionals and health workers in specific areas of mental health, such as counseling skills. Many colleges, for example, send their students to MHNGOs for field placements. Workshops with health workers, teachers and other key groups are a standard feature of the activities of many MHNGOs. Many of these organizations regularly conduct local, national or international conferences, seminars, workshops and symposia to discuss current issues in this field (Kalyanasundaram and Varghese, 2000).[6]

The Richmond Fellowship has successfully established a full two-year MSc program in psychosocial rehabilitation. Two NGOs (Sangath and SCARF) launched a new course (“Leadership in Mental Health”) in 2008 to strengthen skills on scaling up services for people living with mental disorders. The course attracts students from around the world.

### Advocacy and building awareness

Advocating for the needs of under-served and underprivileged sections of the population has been the *raison d’etre* for most MHNGOs. At present, there is very low awareness of the considerable advances in our knowledge of the causes and treatment of mental disorders in India This low awareness, coupled with the enormous stigma attached to mental illness, means that the needs and rights of mentally ill persons are largely ignored. MHNGOs have made raising awareness in different sectors of the community, such as health workers, teachers and lay persons, a priority area. Documentation and dissemination of relevant facts and research, and lobbying policy makers for changes in the law are vital instruments for improving mental healthcare. A prominent example of the success of efforts of MHNGOs is the inclusion of mental disabilities in the disability legislation of the country. The film festival organized by SCARF called the “Frame of Mind,” which features several films portraying mental illness and an international competition for short films on mental health and stigma, is a huge success and has had three editions so far. Similar festivals have since been held in other cities like Kolkata. Many NGOs use short films to spread awareness about their work/cause. Many publish regular newsletters and host web sites marking the close affinity of MHNGOs with contemporary technological advances.

Many MHNGOs adopt methods to enhance the effectiveness of care through empowerment of affected persons and their families. Support groups are widely used as a way to ensure that persons recovering from substance abuse can remain sober. The globally recognized organization, Alcoholics Anonymous, is an example of the kind of support group philosophy which becomes the core to the process of treatment of alcohol dependence. Support groups are also evident in the residential and day care facilities geared to those with severe mental disorders. Some MHNGOs run support groups not for those directly affected by a particular disorder, but for their families. Thus, families of elders with Alzheimer’s disease, adults with schizophrenia and children with autism, meet regularly to discuss common problems, support each other and provide practical solutions to everyday difficulties. Advocacy led by such user and family NGOs may have particularly important impact on government policies. The All India Federation for Mental Health, an umbrella organization of many NGOs working in the field of mental health, and the National Association of the Mentally Ill (NAMI- India) are examples of coalitions of NGOs and consumers respectively which are actively advocating for mental heath policy and care reforms in India.

## MENTAL HEALTH NON-GOVERNMENTAL ORGANIZATIONS STRENGTHS AND LIMITATIONS

Why is it that the MHNGO movement has continued to survive despite the lack of resources and other barriers? This is probably because MHNGOs have some inherent and intrinsic advantages. We can consider the advantages of MHNGOs under three broad categories: Working in Partnership, Innovations in Practice and Transparency in Administration.

- Working in partnerships: One of the great strengths of MHNGOs is their ability to strike up collaborations and partnerships with other agencies or individuals with ease; unlike the public health sector where layers of permissions stifle the scope for collaboration and unlike the private health sector where collaborations may be perceived as a threat to the practice. Most MHGNO activities are provided by multidisciplinary teams of doctors, therapists, health workers, other professionals and volunteers. Partnerships are built not only between medical and non-medical professionals, but also between professionals and families. The close collaboration between academics, clinicians, social workers, rehabilitation workers, remedial teachers, clinical and educational psychologists are a distinct feature which marks MHNGOs as being a very different breed of animal from traditional psychiatric clinics in hospitals or private psychiatry.
- Innovations in practice: MHNGOs are, typically, closer to the community they serve and hence in a better position to be more sensitive to changing needs and perceptions. Further, MHNGO services may be attached with much less stigma than formal psychiatric services, and may thus attract a much wider range of clients. Clinical support, involving diagnosis and treatment of specific mental disorders, is the key to many MHNGO activities. The success of MHNGOs lies in providing services which are accessible, such as through outreach camps, which rely on available human resources, such as the community participatory model of rehabilitation. Many MHNGOs provide a wide range of services which are especially suited for severe and childhood mental health problems. By taking up the process of promoting attitudinal changes in the community and amongst policy makers, MHNGOs also play a key role in advocacy for changes which can benefit all persons with mental illness.
- Transparency in administration: The activities of MHNGOs are driven not by profit but by the desire to achieve a basic quality of care for all clients, irrespective of their ability to pay. They are governed by a relative flexible set of regulations. Employment and promotional avenues can be based on merit as opposed to the traditional governmental holy grail of seniority. Because they are dependent on external funding, MHNGOs are constantly kept on their toes in achieving program objectives and ensuring fiscal accountability. MHNGOs can explore, with remarkable entrepreneurial dynamism, collaborations with any other organization or individual to achieve their objectives.

However, MHNGOs have their fair share of limitations and problems. We can consider these under the broad themes of Sustainability, Accountability and Scope.

- Sustainability: A key problem facing most MHNGOs is the source of their funding, which is largely project-based. The periodic fund raising required to augment resources can take up a good deal of time and energy. Staff members have no guarantee of employment beyond a defined project period. As a consequence, some MHNGOs suffer a high turn over of staff. This is partly because staffs are appointed on specific funded projects and their continuity depends on the funding available. There might be a temptation to dilute goals and objectives as a response to availability of funding. Donor funding is notoriously fickle; priorities change over time, and MHNGOs often reinvent their objectives to keep afloat. The recent trend for massive investment in HIV/AIDS related work, though important in its objectives, is concentrating the bulk of donor money to this one-disease issue. Many MHNGOs and, indeed, some MHNGOs are adding HIV/AIDS as core priorities to secure these funds. While this may broaden the scope of MHNGOs by enabling an integration of existing priorities with new ones, there is equally a need not to allow the focus on mental health to be diluted to the point that it becomes irrelevant
- Accountability: Some MHNGOs have poorly established mechanisms for evaluation and monitoring. Although networking is actively sought for project collaboration, there is no similar zeal for review and monitoring from external assessors. There has been considerable public concern regarding the misuse of funds and lack of financial accountability of NGOs in general. Although this may not be as significant an issue in the context of MHNGOs where funds are scarce, MHNGOs would be well advised to ensure transparency in accounting for their funds. As MHNGOs become larger and more professionalized, there is a danger of increasing bureaucratization with increasing administrative costs. MHNGOs should be wary of this from the beginning since it could well dampen creativity and flexibility, two elements which give MHNGOs their unique flavor.
- Scope: Finally, and perhaps the most important limitation is the limited scope of individual MHNGOs. The world of most MHNGOs is confined to a city or a few villages. There is, however, a need to transplant the wide experience of these onto a larger canvas, ideally through influencing policies and programs for the entire state and country. For changes to occur on this wider canvas there is little doubt that the public or government health sector must play a key and leading role. MHNGOs can, in this context, be seen as innovators who develop locally relevant models which can then be implemented on a national scale.

## CONCLUSION

MHNGOs have made tremendous strides in mental health promotion and care, against massive odds ranging from low awareness about mental illness to lack of motivation donors. Although there can be little dispute whether the MHNGOs have a definite role to play in meeting mental health needs in India, there is also little doubt that their impact on mental health care at the national level has been marginal. For example, there are very few MHNGOs working in rural or impoverished areas. The strength of MHNGOs does not lie in their ability to reach out to the millions of persons with mental disorders, but in evolving and perfecting quality programs and models which have the character of replicability. Through innovation and accountability, MHNGOs can provide models for the public healthcare system to emulate and partner. However, they cannot entirely meet the needs of the under-served and underprivileged sectors of our population. That responsibility, was, is, and must rest principally with the public health sector. We believe that the time and setting is right not only for the emergence of new MHNGOs, but also for the consolidation and strengthening of existing ones.

We specifically call for the inclusion of MHNGOs as full partners of the government services in the National Mental Health Program. Such a partnership could take several forms.

1. Participation in intersectoral committees to monitor and implement the NMHP in each district, involving government, NGO and psychiatric stakeholder representation. These committees can be empowered to take on roles of combating stigma, supporting user groups, monitoring rights, and capacity building.
2. NGOs running DMHP following the model of NGOs adopting primary health centers in some districts; here NGOs become providers of the DMHP services, especially in districts where government mental health services are weak.
3. NGOs developing niche community based services including day care centers and residential facilities for chronically disabled patients or children or mental health promotion activities, help lines for distressed suicidal patients, facilitating user and family support groups and assisting with livelihoods and employment generation and so on.
4. NGO placements becoming mandatory for psychiatric training for doctors and nurses whose current training programs are mainly hospital based, thereby missing out on the entire range of community-based and PSR experiences.
5. NGO representation should be sought in all committees, task forces involved with planning of mental health activities and program implementations at the state and national levels.

We urge the government agencies to take note of the huge public health implications of mental disorder and the lack of organized services for the mentally ill, and provide support for MHNGOs in the ways proposed above. Given a favorable climate, we are sure that the MHNGO movement in Mental Health will not be a sporadic or isolated phenomenon as it is now, but a more enduring and unified force in the realm of Mental Health in India.
